# Maternal serum albumin in the late trimester and birth weight: a cross-sectional study from Jiangxi, China

**DOI:** 10.1371/journal.pone.0319494

**Published:** 2025-04-15

**Authors:** Jun Xiong, Huan Li, Jian-Min Zhang, Shi-Qi Li, Chang Ge, Hui-Xin Zheng, Xiao-Qing Tan, Jia-Wei Zheng, Xiao-Ju He, Shu-Ying Zhu

**Affiliations:** 1 The Second Affiliated Hospital of Nanchang University, NanChang, China; 2 Nanchang University, Nanchang, China; 3 The Second Clinical Medical College of Nanchang University, Nanchang, China; King Saud University / Zagazig University, EGYPT

## Abstract

**Background:**

The links of maternal serum albumin (ALB) concentration in the late trimester and infant birth weight remain equivocal. Accordingly, we focused on the investigation of the correlation of maternal serum albumin concentration and infant birth weight among women during pregnancy in Jiangxi, China.

**Methods:**

1214 subjects were recruited for the present cross-sectional study. Infants of low birth weight (LBW) had a weight <2500g when they are born. Albumin was categorized as <30, 30-<35 and ≥35 g/L, with a concentration of <30g/L indicating hypoproteinemia. Low birth weight and the correlations of maternal serum albumin concentration in the late trimester and infant birth weight were evaluated binary logistic regression analyses and linear regression on the basis of multiple variables, separately.

**Results:**

The overall prevalence of hypoproteinemia was 7.83%. Maternal serum albumin concentration in the late trimester was positively correlated to infant birth weight (β, 0.03; 95% confidence interval [CI]: 0.02, 0.04), as indicated by multivariate linear regression analyses. Besides, a negative correlation of maternal serum albumin concentration in the late trimester and low birth weight (odds ratio [OR], 0.84; 95 percent CI: 0.78, 0.91) was reported through multivariable binary logistic regression analyses, which showed consistency with the above result. In comparison to individuals in ALB < 30 g/L group of maternal serum albumin, the adjusted β and OR values of albumin for infant birth weight and low birth weight were 0.40 (95 percent CI: 0.26, 0.54) and 0.18 (95 percent CI: 0.09, 0.39), separately. Results of smoothing curve fitting confirmed the linear correlation of maternal serum albumin and infant birth weight and low birth weight. Maternal serum albumin and infant birth weight were consistent in the subgroups below: smoking habit, antenatal visits, sex of the newborn, education, maternal age, parity, hemoglobin, pre pregnancy body mass index (BMI) and gestation age at delivery.

**Conclusion:**

A higher maternal serum albumin in the late trimester is associated with a lower risk of infant birth weight. The data suggests that maternal serum albumin in the late trimester may serve as a simple and effective tool for the assessment of the low birth weight risk in clinical practice.

## Introduction

From pregnancy to delivery, women during pregnancy undergo a series of physiological and biochemical changes [1]. Women during pregnancy should not only provide their own nutritional needs, but also provide the nutrition needed for fetal growth and development [2]. Accordingly, the nutritional status of women during pregnancy is related to their own health, while it affects fetal development and infant birth weight [2,3]. Albumin (ALB) possesses a long half-life and a large pool in the circulation, and the serum concentration has been widely tested in clinical sites for the evaluation of long-term variations in protein nutritional status [4,5]. Low birth weight (LBW) acts as a critical risk factor for infant morbidity and mortality [6]. No consistent conclusion has been drawn by existing research on the correlation of maternal serum ALB and birth weight. Limited data suggest that serum reduced ALB ratio in the third trimester displayed a positive correlation to birth weight [5]. However, Maher et al. [7] revealed that there was no pronounced correlation of ALB levels and birth weight. Moreover, there has been scarce research on the linearity of serum ALB concentration and infant birth weight. A hypothesis was proposed in this study, i.e., the correlation of maternal serum ALB and birth weight may change in ethnic groups, and their correlation may be a linear correlation. Accordingly, we investigated the correlation of maternal serum ALB and infant birth weight in Jiangxi, China.

## Methods

### Ethics statement

The Second Affiliated Hospital of Nanchang University’s ethics review boards issued the approval for our work, and we followed the Declaration of Helsinki. We recruited the subjects signing informed consent. We adopted fingerprinting when subjects failed to write. The process here gained approval from the ethics committee.

### Study population

We carried out a cross-sectional survey in two types of unit of the Second Affiliated Hospital of Nanchang University in Jiangxi, China (i.e., delivery units (DU) and the antenatal care units (ANC)). We screened and recruited these subjects from women during pregnancy who had an age of ≥18 went to DU and attended ANC routine care in a range between May 2022 and June 2023.

### Study enrolments

**Fig 1:**
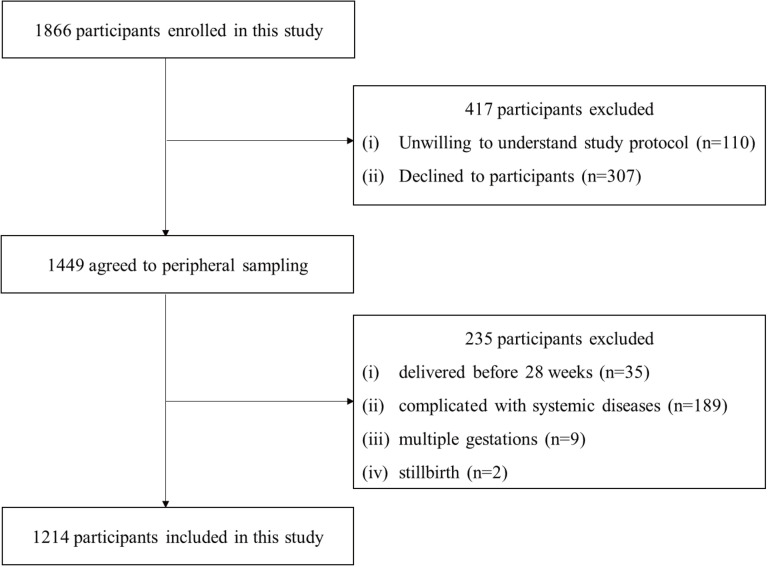
Flow chart of study participants.

We referred and screened 1866 pregnant/delivering female subjects in DU. Of the above-mentioned 1756, a tendency to be informed about this study protocol was observed. Specifically, 1449 gave consent and showed agreement to peripheral sampling, whereas 307 refused the involvement. Technical staff who had received special training interviewed 1449 recruited subjects while collected data (e.g., weight at birth, obstetric complications in the pregnant period and sociodemographic characteristics). When subjects performing delivery before 28 weeks (n = 35) were excluded, including subjects suffering from systemic diseases, i.e., stillbirth (n = 2), diabetes mellitus, haematological diseases, liver diseases, renal failure and pregnancy related hypertensive disease (n = 189), and multiple gestations (n = 9), we covered 1214 subjects in the final investigation (Fig 1). All blood samples were collected in the third trimester before delivery.

### Study definitions for birth weight and maternal variables

Based on the date of the last menstruation, the gestational age was evaluated. However, if there was any uncertainty or doubt regarding the estimation of gestational age and fetal positioning, we confirmed the ages using ultrasound measurements of the gestational sac size and the embryo length. Infants with LBW possessed a weight <2500g when they were born. In line with the regulations of the World Health Organization, hemoglobin fell into four groups [8]: ≥110, 100–109, 70–99, and <70 g/L. The 1214 women during pregnancy fell into three groups [9,10]: (i) ALB < 30 g/L (n = 95), (ii) ALB 30-<35 g/L (n = 604), and (iii) ALB ≥ 35 g/L (n = 515). ALB levels lower than 30g/L were considered indicative of hypoalbuminemia.

### Statistical analysis

Continuous variables were expressed as means (SD), and categorical variables were expressed as ALB concentration (i.e., <30, 30-<35, ≥35 g/L) and overall-subject percentages. We tested the normality of continuous variables and then log-transformed the variables, when required. We performed the chi-square test to investigate categorical variables. We employed multivariable binary logistic regression and multivariable linear regression to analyze ALB concentration and the correlations of birth weight and LBW, separately. We confirmed that regression analysis’s applicable conditions were qualified. Next, the results had the expression of β coefficients and odds ratios (ORs) that possessed confidence intervals of 95% (CIs). A total of three sets of models were developed in accordance with relevant literature and clinical experiences. In the regression analysis models, variables were selected due to their clinical importance, statistical significance in the univariable analysis, and the potential confounders effect estimates that individually changed by at least 10%. Model 1 was subjected to the control regarding none. Model 2 was subjected to the control regarding maternal age and education. Model 3 was subjected to the control regarding variable in model 2 plus weight gain during pregnancy, parity, maternal smoking, pre pregnancy body mass index (BMI), the number of antenatal visits, sex of the newborn, gestational age of delivery, as well as total serum protein and hemoglobin concentration. Based on all covariates from model 3, we tested the multicollinearity, and all variables conformed to the criteria. The multicollinearity test used variance inflation factor, which was less than or equal to five, indicating that there was no multicollinearity. We carried out subgroup analysis based on the number of prenatal examinations, smoking, sex of the newborn, pre pregnancy BMI, gestational age of delivery, parity, hemoglobin concentration, education, and maternal age, with the aim of testing potential confounder-ALB concentration interactions. Specifically, we incorporated cross-product interaction terms into the relevant multiple linear regression models for the above interaction test. Dose–response correlation of birth weight and LBW and ALB concentration were evaluated by employing a fitted smoothing curve (penalized spline method) and a generalized additive model (GAM). Furthermore, we carried out stratified analyses and interaction testing to evaluate probable modification of the correlation with birth weight.

Statistical analyses were conducted by using R statistical package (http://www.r-project.org) and Empower (R) software (www.empowerstats.com). We set a two-tailed *P* <  0.05 as statistical significance.

## Results

### Characteristics of subjects

Table 1 presents the basic characteristics of the 1214 subjects categorized according to maternal serum ALB concentration. 28.7 (4.6) years was obtained as the mean (SD) age of the subjects. 7.83% was calculated as the total prevalence of hypoalbuminemia, and nearly 43.2% of women during pregnancy with hypoalbuminemia have preterm birth. In comparison to women during pregnancy with hypoalbuminemia, subjects with greater ALB concentrations possessed higher gestational weight gain, longer duration of gestation and lower parity (all *P* < 0.05). In the ALB < 30 g/L group, the proportion of women during pregnancy with less than 5 times of antenatal visits was 24.2%, pronouncedly exceeding 7% and 11.1% in ALB 30- < 35 g/L group and ALB ≥ 35 g/L group (*P* <  0.05). The infant birth weight was indicated to increase with rising ALB concentration. In the ALB < 30 g/L group, LBW took up 41.1%, whereas the proportion decreased by 11.1% and 8% in the ALB 30- < 35 g/L group and ALB ≥ 35 g/L group, respectively (*P* < 0.05). We did not report any pronounced differences in the three groups regarding the maternal smoking habit, education levels, pre pregnancy BMI and the sex of the newborn (all *P* >  0.05).

**Table 1:** Baseline characteristics of participants stratified by maternal serum ALB in the late trimester.

Characteristics	Total	Maternal serum ALB (g/L)			*P*- value
		<30	30-<35	≥ 35	
**N**		95	604	515	
**serum total protein (g/L)**	62.9 ± 5.8	55.1 ± 6.6	61.3 ± 4.8	66.3 ± 4.2	<0.001
**pre pregnancy weight (kg)**	53.8 ± 7.7	52.2 ± 8.2	54.0 ± 7.6	53.9 ± 7.7	<0.001
**gestational weight gain (kg)**	12.6 ± 3.8	11.2 ± 3.7	12.3 ± 3.9	13.1 ± 3.7	<0.001
**Maternal age (year), n (%)**					0.001
**<**25	293 (24.1%)	18 (18.9%)	121 (20.0%)	154 (29.9%)	
25–34	785 (64.7%)	66 (69.5%)	405 (67.1%)	314 (61.0%)	
≥35	136 (11.2%)	11 (11.6%)	78 (12.9%)	47 (9.1%)	
**Education**					0.068
No formal education	217 (17.9%)	25 (26.3%)	114 (18.9%)	78 (15.1%)	
Primary	549 (45.2%)	43 (45.3%)	267 (44.2%)	239 (46.4%)	
Secondary/University +	448 (36.9%)	27 (28.4%)	223 (36.9%)	198 (38.4%)	
**pre pregnancy BMI**					0.471
<18.5	188 (15.5%)	20 (21.1%)	90 (14.9%)	78 (15.1%)	
18.5–24	935 (77.0%)	70 (73.7%)	471 (78.0%)	394 (76.5%)	
>24	91 (7.5%)	5 (5.3%)	43 (7.1%)	43 (8.3%)	
**Smoking habit, n (%)**					0.190
Yes	51 (4.2%)	5 (5.3%)	19 (3.1%)	27 (5.2%)	
No	1163 (95.8%)	90 (94.7%)	585 (96.9%)	488 (94.8%)	
**Antenatal visits, n (%)**					<0.001
0–4	122 (10.0%)	23 (24.2%)	42 (7.0%)	57 (11.1%)	
≥5	1092 (90.0%)	72 (75.8%)	562 (93.0%)	458 (88.9%)	
**Hemoglobin (g/L), n (%)**					<0.001
<70	28 (2.3%)	15 (15.8%)	8 (1.3%)	5 (1.0%)	
70 – 99	335 (27.6%)	41 (43.2%)	186 (30.8%)	108 (21.0%)	
100 – 109	279 (23.0%)	19 (20.0%)	161 (26.7%)	99 (19.2%)	
≥ 110	572 (47.1%)	20 (21.1%)	249 (41.2%)	303 (58.8%)	
**LBW, n (%)**					<0.001
Yes	147 (12.1%)	39 (41.1%)	67 (11.1%)	41 (8.0%)	
No	1067 (87.9%)	56 (58.9%)	537 (88.9%)	474 (92.0%)	
**Parity**					0.009
0	573 (47.2%)	44 (46.3%)	257 (42.5%)	272 (52.8%)	
1–2	595 (49.0%)	46 (48.4%)	326 (54.0%)	223 (43.3%)	
≥3	46 (3.8%)	5 (5.3%)	21 (3.5%)	20 (3.9%)	
**gestation age at delivery (weeks), n (%)**					<0.001
28–33 + 6	43 (3.5%)	19 (20.0%)	13 (2.2%)	11 (2.1%)	
34–36 + 6	99 (8.2%)	22 (23.2%)	51 (8.4%)	26 (5.0%)	
≥37	1072 (88.3%)	54 (56.8%)	540 (89.4%)	478 (92.8%)	
**sex of the newborn, n (%)**					0.603
Male	673 (55.4%)	48 (50.5%)	338 (56.0%)	287 (55.7%)	
Female	541 (44.6%)	47 (49.5%)	266 (44.0%)	228 (44.3%)	

Data are presented as n (percentage), mean ±  standard deviation.

### Correlation of infant birth weight, LBW and maternal serum ALB concentration

**Fig 2:**
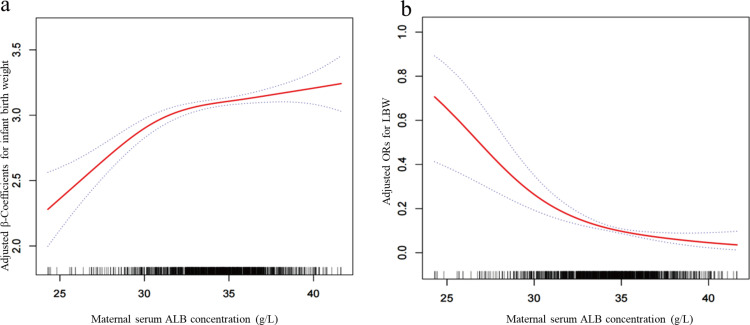
Dose-response relationship of maternal serum ALB in the late trimester with infant birth weight (a) and LBW (b). Adjusted for smoking habit, antenatal visits, serum total protein, sex of the newborn, pre pregnancy weight, education, gestational weight gain, maternal age, parity, anemia, pre pregnancy BMI and gestation age at delivery.

In general, the smoothing curve indicated a pronounced linear correlation of maternal serum ALB concentration and infant birth weight and the LBW risk (Fig 2a and b). Regarding 1 unit increment in serum ALB, the infant birth weight varied by 0.03 (95 percent CI: 0.02, 0.04) in line with the estimated result based on regression β coefficients. Furthermore, 0.84 was computed as the OR for the LBW risk (95 percent CI: 0.78, 0.91).

Table 2 shows that all models (models 1–3) adjusted in terms of potential confounders showed a positive correlation of serum ALB and infant birth weight. If serum ALB was categorized into three groups, the adjusted β of ALB in the final adjusted model (model 3) on infant birth weight regarding subjects in ALB 30- <  35 g/L group and ALB ≥ 35 g/L group reached 0.37 (95 percent CI: 0.25, 0.49) and 0.40 (95 percent CI: 0.26, 0.54), separately, in comparison to the data in the ALB < 30 g/L group (*P* for trend <0.001). In Table 3, compared to subjects in the ALB < 30 g/L group, the adjusted ORs of ALB on LBW regarding subjects in the ALB 30- < 35 g/L group and ALB ≥ 35 g/L group reached 0.26 (95 percent CI: 0.14, 0.49) and 0.18 (95 percent CI: 0.09, 0.39), separately. Furthermore, the *P* was pronounced for trend in the overall models, which suggested a dose–response correlation of serum ALB and infant birth weight and LBW.

**Table 2:** Association of maternal serum ALB in the late trimester with infant birth weight in different models.

Maternal serum ALB	Infant birth weight, β(95% CI)		
	Model 1	Model 2	Model 3
**Continuous**	0.03 (0.02, 0.04)	0.03 (0.02, 0.04)	0.03 (0.02, 0.04)
**Categories**			
**<30** g/L	0.00	0.00	0.00
**30-** **<** **35** g/L	0.54 (0.42, 0.65)	0.52 (0.41, 0.64)	0.37 (0.25, 0.49)
≥ **35** g/L	0.57 (0.46, 0.69)	0.56 (0.45, 0.68)	0.40 (0.26, 0.54)
***P* for trend**	< 0.001	< 0.001	< 0.001

Model 1: adjusted none

Model 2: adjusted maternal age, education

Model 3: adjusted smoking habit, antenatal visits, serum total protein, sex of the newborn, pre pregnancy weight, education, gestational weight gain, maternal age, parity, anemia, pre pregnancy BMI and gestation age at delivery.

**Table 3:** Association of maternal serum ALB in the late trimester with LBW in different models.

Maternal serum ALB	LBW, OR (95% CI)		
	Model 1	Model 2	Model 3
**Continuous**	0.83 (0.78, 0.87)	0.83 (0.79, 0.88)	0.84 (0.78, 0.91)
**Categories**			
**< 30** g/L	1.00	1.00	1.00
**30- <** **35**g/L	0.18 (0.11, 0.29)	0.19 (0.11, 0.30)	0.26 (0.14, 0.49)
≥ **35** g/L	0.12 (0.07, 0.21)	0.13 (0.08, 0.22)	0.18 (0.09, 0.39)
***P* for trend**	< 0.001	< 0.001	< 0.001

Model 1: adjusted none

Model 2: adjusted maternal age, education

Model 3: adjusted smoking habit, antenatal visits, serum total protein, sex of the newborn, pre pregnancy weight, education, gestational weight gain, maternal age, parity, anemia, pre pregnancy BMI and gestation age at delivery.

### Subgroup analyses by potential effect modifiers

**Fig 3:**
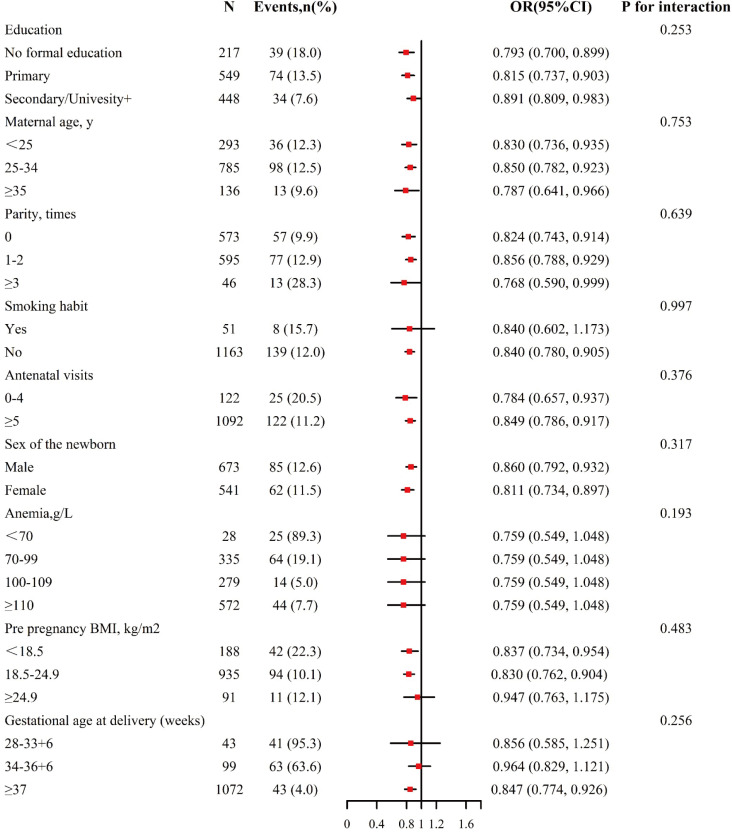
Subgroup analyses of the effect of maternal serum ALB in the late trimester on infant birth weight. Each subgroup analysis adjusted for smoking habit, antenatal visits, serum total protein, sex of the newborn, pre pregnancy weight, education, gestational weight gain, maternal age, parity, anemia, pre pregnancy BMI and gestation age at delivery.

Stratified analyses were carried out for the evaluation of the influence exerted by serum ALB (per 1 unit increment) on infant birth weight within a range of subgroups (Fig 3). The infant birth weight-serum ALB correlation was consistent in the subgroups below (all *P* for interaction >0.05): gestation age at delivery (28–33 ^+^ ^6^, 34–36 ^+^ ^6^, ≥ 37 weeks), pre pregnancy BMI (<18.5, 18.5–24.9, ≥ 24.9 kg/m^2^), anemia (<70, 70–99, 100–109, ≥  110 g/L), parity (0, 1–2, ≥ 3 times), maternal age ( < 25, 25–34, ≥  35 y), education (no formal education, primary, secondary/university+), sex of the newborn (male vs. female), antenatal visits (0–4 vs. ≥  5), and smoking habit (no vs. yes).

## Discussion

In this study, the result indicated that maternal serum ALB concentration in third trimester showed an independent correlation to infant birth weight. Maternal serum albumin concentration was positively associated with infant birth weight, and a higher maternal serum albumin was associated with a lower risk of infant birth weight. Moreover, the correlations of maternal serum ALB and infant birth weight were consistent and no pronounced interactions were reported in the subgroup below: maternal education, age, parity, smoking habit, antenatal visits, sex of the newborn, anemia, pre pregnancy BMI, as well as gestational age at delivery (all *P* for interaction >  0.05).

Protein is the basic material of the fetus, placenta, uterus and other tissues [11,12]. During pregnancy, the protein synthesis and protein breakdown in the body increase of pregnant woman, whereas they generally possess a positive nitrogen balance [13]. Under the effect of blood dilution during pregnancy, plasma protein begins to be reduced from early pregnancy, mainly manifested as a decrease in ALB [9,13]. ALB refers to a spherical peptide chain that is comprised of 585 amino acids, which possesses a molecular weight of 66kDa [9]. The half-life of serum ALB in women during pregnancy is 21 days. Moreover, its concentration acts as a typical marker of (protein energy) malnutrition, which is likely to mirror the quality of diet [5,14]. The above-mentioned observations were consistent with our finding that no pronounced difference existed in serum ALB concentration in third trimester among different pre pregnancy BMI groups. In the second trimester, a greater diet quality can facilitate a preterm birth reduction, as indicated by a recent prospective cohort study [15]. Some scholars also highlighted that maternal serum can reduce ALB ratio in the third trimester will display a positive correlation to gestation period length [4,5]. The possible reason for the above result is that ALB is a low affinity, high capacity carrier of endogenous and exogenous compounds in the human body [16–18]. The decrease in ALB concentration directly triggers a decrease in bound calcium in the serum [9], whereas the concentration of ionic calcium, which facilitates uterine fiber contraction, increases, which results in shortened gestational weeks [19]. This study found that women during pregnancy in the hypoalbuminemia group have shorter gestation period length while possessing pronouncedly fewer antenatal visits and less weight gain during pregnancy than those with normal ALB. The reason for the above result is that women during pregnancy who undergo frequent antenatal visits can receive more guidance from doctors on their dietary structure, leading to a gradual increase in pregnancy weight as the gestational age increases.

Serum ALB, a vital indicator of protein intake and nutritional status in women during pregnancy [4,5], is also a research hotspot in its impact on infant birth weight. Wada et al. [5] investigated 229 Japanese women during pregnancy and found that serum reduced ALB ratio in the third trimester displayed a pronounced and positive correlation to infant birth weight, which would be mediated by maternal protein nutritional status. As revealed by the further study, maternal energy and protein intake showed a pronounced positive correlation to birth weight [20–22], and a low protein diet during pregnancy triggers the occurrence of LBW [23–25]. The above finding is consistent with ours, i.e., maternal serum ALB was positively correlated to infant birth weight. The higher the ALB level of women during pregnancy, the lower the LBW risk, and this trend was statistically significant. We believe that there are three reasons for this phenomenon. First, ALB can maintain the constant osmotic pressure of plasma colloid [26–28] and ensure sufficient blood supply of uterus and placenta. Second, ALB, a non-specific transport protein [29–31], is capable of transporting different nutrients from the mother to the fetus and promote its growth and development. Third, as mentioned previously, the reduction of ALB concentration is easy to shorten gestational age, and gestational age showed a positive correlation to birth weight [32–34], such that there might be a close correlation of birth weight and ALB level. However, some scholars have reached different conclusions on the correlation of maternal ALB and infant birth weight. Maher et al. [7] obtained serum samples from 289 indigent multiparous women, confirming that there was no pronounced correlation of ALB levels at 30 weeks and birth weight. An observational study carried out by Chong et al. in Singapore determined the macronutrient intake of women during pregnancy by employing a dietary recall for 14 h and a food diary for 3 days and confirmed that maternal protein intake in the pregnant course does not display any correlations to a multiethnic Asian population’s offspring birth weight [35]. Socioeconomic status, lifestyle behaviors, sample size, and various race backgrounds could account for the discrepancy.

This study possesses some strengths, which are comprised of the large-sample-size study design. The present study has evaluated the potential correlation of maternal serum ALB and infant birth weight in Jiangxi, China for the first time. Moreover, this study covered women during pregnancy conforming to the criteria of being included throughout the year. Some adjustments were carried out for minimizing residual confounders. ALB concentration was set as two types of variables (categorical and continuous), with the aim of reducing the contingency in the data analysis. Furthermore, we performed the subgroup analyses.

Nevertheless, several limitations should be considered. First, this cross-sectional survey failed to illustrate the causal relationship of infant birth weight and maternal serum ALB. Second, our study did not examine the influence of albumin levels during the first and second trimesters of pregnancy on infant birth weight. Third, subjects in this study were recruited from Jiangxi in southern China, such that the findings here turned out to be less generalizable to other populations. Lastly, notwithstanding the consideration of factors as many as possible, other potential confounding factors (Sleep time of women during pregnancy, work-time physical activity intensity, iron supplement) might remain not be covered.

## Conclusion

The result achieved in this study confirmed that a linear positive correlation of infant birth weight and the maternal serum ALB was reported in women during pregnancy in the third trimester from Jiangxi, China. The above-mentioned correlation was not determined by demographics and other risk factors. Knowledge of maternal serum ALB concentration can be considered a preventive marker to identify LBW, as indicated by the above-mentioned findings. Furthermore, it is imperative to conduct on in-depth longitudinal epidemiological research for evaluating the effects exerted by the infant birth weight and the maternal serum ALB.

### Clinical perspectives

- Albumin (ALB) can reflect the nutritional status of pregnant women, and the nutritional status of pregnant women plays an extremely important role in fetal growth and infant birth weight.
- In our study, we found that maternal serum ALB concentration in third trimester was independently associated with infant birth weight. Maternal serum ALB was positively correlated with infant birth weight, but negatively correlated with low birth weight (LBW).
- Maternal serum ALB may serve as a simple and effective tool for the assessment of the risk of LBW in clinical practice.

## Abbreviations

ALB: albumin
LBW: low birth weight
GAM: Generalized additive model
ANC: antenatal care units
DU: delivery units
SD: standard deviation
OR: odds ratio
CI: confidence interval
BMI: body mass index
